# Modulation of Illusory Auditory Perception by Transcranial Electrical Stimulation

**DOI:** 10.3389/fnins.2017.00351

**Published:** 2017-06-20

**Authors:** Giulia Prete, Anita D'Anselmo, Luca Tommasi, Alfredo Brancucci

**Affiliations:** Department of Psychological Science, Health and Territory, Università degli Studi “G. d'Annunzio” Chieti – PescaraChieti, Italy

**Keywords:** Deutsch's illusion, auditory cortex, transcranial direct current stimulation (tDCS), transcranial random noise stimulation (tRNS), acoustic stimuli

## Abstract

The aim of the present study was to test whether transcranial electrical stimulation can modulate illusory perception in the auditory domain. In two separate experiments we applied transcranial Direct Current Stimulation (anodal/cathodal tDCS, 2 mA; *N* = 60) and high-frequency transcranial Random Noise Stimulation (hf-tRNS, 1.5 mA, offset 0; *N* = 45) on the temporal cortex during the presentation of the stimuli eliciting the Deutsch's illusion. The illusion arises when two sine tones spaced one octave apart (400 and 800 Hz) are presented dichotically in alternation, one in the left and the other in the right ear, so that when the right ear receives the high tone, the left ear receives the low tone, and vice versa. The majority of the population perceives one high-pitched tone in one ear alternating with one low-pitched tone in the other ear. The results revealed that neither anodal nor cathodal tDCS applied over the left/right temporal cortex modulated the perception of the illusion, whereas hf-tRNS applied bilaterally on the temporal cortex reduced the number of times the sequence of sounds is perceived as the Deutsch's illusion with respect to the sham control condition. The stimulation time before the beginning of the task (5 or 15 min) did not influence the perceptual outcome. In accordance with previous findings, we conclude that hf-tRNS can modulate auditory perception more efficiently than tDCS.

## Introduction

The effects of transcranial Electrical Stimulation (tES) have been widely exploited in the last decade to investigate the causal relationship between cortical activity of specific brain areas and cognitive or perceptual tasks (e.g., Miniussi and Ruzzoli, 2013; Filmer et al., 2014). Transcranial direct current stimulation (tDCS) is a type of tES that modulates cortical excitability in a polarity-dependent manner. During the stimulation, the current is direct and flows from an active to a reference electrode, inducing a polarization of cortical neurons at a subthreshold level (Miniussi et al., 2013). The effects of tDCS depend on the current polarity: anodal stimulation typically induces a cellular membrane depolarization and cathodal stimulation determines a hyperpolarization (Nitsche and Paulus, 2000; Nitsche et al., 2003). In a realistic head model the current is oriented toward the closest conducting brain area, but it can reach also distant regions with respect to the target site, even if the median current density tends to decrease with increasing distance from the electrodes (Wagner et al., 2013). Anodal and cathodal stimulations produce respectively a facilitation and an inhibition of neural processing (Antal et al., 2004; Fregni et al., 2005). A different type of current release characterizes transcranial random noise stimulation (tRNS). It consists in the application of repetitive alternating current over the cortex at random frequencies (0.1–640 Hz). Through the application of tRNS at high frequency (100–640 Hz) and with an intensity equal to or >1 mA, it has been shown that the stimulation is able to positively modulate the excitability of motor and auditory areas (Moliadze et al., 2012; Van Doren et al., 2014) as well as to improve performance in behavioral tasks, for example in the domain of motor and visual perception learning (Terney et al., 2008; Fertonani et al., 2011).

Despite the bulk of studies investigating the effects of tES in the motor (Sehm et al., 2013; Inukai et al., 2016), visual (Accornero et al., 2007; van der Groen and Wenderoth, 2016) and cognitive domain (Heimrath et al., 2012; Dormal et al., 2016), relatively few studies have explored tES effects in the auditory modality (for a recent review see Heimrath et al., 2016). Moreover, some studies evidenced the potential of tDCS to alter neuronal excitability in the auditory cortex (AC; Zaehle et al., 2011). Effects of tDCS have also been found in auditory perceptual processing. For instance, anodal stimulation over AC improves auditory temporal resolution abilities (Ladeira et al., 2011). tDCS also interferes with pitch discrimination, mainly during the stimulation of the right rather than the left Heschl's gyrus (Mathys et al., 2010; Tang and Hammond, 2013; Matsushita et al., 2015) and it enhances mismatch negativity response during the presentation of tones with deviant frequencies (Impey and Knott, 2015). Further, tRNS applied at high frequency (101–640 Hz) was found to induce increased excitability in AC, as measured with EEG auditory steady-state responses (Van Doren et al., 2014).

Considering the results obtained in the modulation of AC excitability and the possibility to interfere with auditory perception, in the present study we intended to shed more light on the domain of AC stimulation and auditory perception. To this aim, we decided to use an acoustic sequence eliciting the Deutsch's illusion (also called “Octave illusion;” Deutsch, 1974a) in which, starting from an identical stimulus, subjects can experience different auditory percepts. The illusion is composed of a sequence of dichotic tones, alternating in frequency typically between 400 and 800 Hz (Brancucci et al., 2009) and presented repeatedly and in alternation, so that when the right ear receives the high tone, the left ear receives simultaneously the low tone and vice versa. Most listeners report perceiving a single high-pitched tone in one ear alternating with a single low-pitched tone in the other ear (see **Figure 2**).

Studies investigating the neural bases of the illusion demonstrated that when the same acoustic stimulus is perceived in different ways it produces brain activations which vary along with the perception of two dimensions, i.e., pitch, a high or a low tone, and side, a tone perceived at the left or right ear (Brancucci et al., 2011, 2016). Given that the perception of the illusion, and in particular of the pitch, involves the activation of a bilateral network including the Heschl's gyrus, we wanted to test whether the application of tES on AC could lead to a different perception of the illusion. Specifically, we hypothesized that the hyper-activity of the temporal cortex induced by tES could interfere with auditory processing, making more difficult the emergence of illusory percepts (Ross and Tervaniemi, 1996; Brancucci et al., 2016). In a first study we evaluated the effects of anodal and cathodal tDCS, starting from polarity specific effects which were found with AC stimulation (Joos et al., 2014): we used an active electrode located on the left or right temporal cortex and a non-cephalic reference electrode, in order to eliminate potential confounding effects of the reference electrode. In this study we hypothesized that anodal and cathodal tDCS may respectively favor and prevent the veridical perception of the auditory sequence normally eliciting the Deutsch's illusion. Furthermore, in a second study, we applied hf-tRNS on the temporal cortex with a bilateral temporal montage, given that tRNS has no current directionality. In both studies, we stimulated before and during the presentation of the acoustic stimuli (online) because different results showed that online stimulation is more effective in inducing facilitating effects than offline stimulation, during perceptual tasks (e.g., Stagg et al., 2011; Pirulli et al., 2013).

We expected to observe a modulation of the subjective perception of the illusion: the main hypothesis was that tES applied over the temporal cortex could improve the (veridical) processing of the tone sequence (pitch/ear), hampering the perception of the illusion. Furthermore, we expected to find that the perception of the illusion would be differently affected in relation to the different type of stimulation (anodal tDCS, cathodal tDCS, tRNS). In particular, starting from the results of previous studies, showing that during a verbal dichotic listening paradigm hf-tRNS applied on the AC bilaterally enhances the well-known right ear advantage with respect to sham stimulation (Prete et al., under review), whereas unilateral tDCS does not have effects on it (D'Anselmo et al., 2015), we predicted bilateral tRNS to be more efficient than unilateral tDCS in modulating auditory perception. Due to the different montages used in the present studies (i.e., unilateral montage in the tDCS study and bilateral montage in the tRNS study), we could not directly compare the effects of the two stimulation setups, but we aimed at investigating the possible effects of two different techniques and two different electrode montages on the Deutsch's illusion, starting from the scarcity of evidence in this field.

## Experiment 1 (tDCS)

### Materials and methods

#### Participants

A sample of 60 healthy volunteers took part in the study. Subjects were randomly divided into two subgroups: 30 participants were assigned to the cathodal group (15 females, mean age = 21.67 ± 0.65), and 30 participants were assigned to the anodal group (15 females, mean age = 20.33 ± 0.19). Handedness scores were calculated using the Edinburgh Handedness Inventory (Oldfield, 1971), according to which the handedness score ranges from −100 (totally left handed) to +100 (totally right handed). For the first group (cathodal stimulation) the mean value of the handedness scores was 44.36 (±1.42), including three left-handers (scores <0). For the second group (anodal stimulation) the mean value of the handedness scores was 44.43 (±1.4), including three left-handers.

Participants were enrolled if they did not show auditory impairments and no different hearing thresholds (±5 dBA) between left and right ears, as measured by an audiometric functional assessment (Brancucci et al., 2005). All participants were free from any history of psychiatric or neurological disorders, had normal or corrected-to-normal vision and no implanted metal objects. The whole procedure was carried out in accordance with the principles of the Declaration of Helsinki, the protocol was approved by the Biomedical Research Ethics Committee, University of Chieti-Pescara, and participants gave written and informed consent before beginning the experiment.

#### tDCS and general procedure

tDCS was delivered by a battery-driven, constant current stimulator (DC-Stimulator, NeuroConn GmbH, Germany) through a pair of surface saline-soaked sponge electrodes (5 × 7 cm, area: 35 cm^2^) kept firm by elastic bands. A constant current of 2 mA was applied for 20 min, according to safety guidelines (Poreisz et al., 2007) with a ramping period of 60 s both at the beginning and at the end of the stimulation.

The active electrode was placed between C3/4 and T3/4 sites (specifically C5 and C6 sites) of the 10–20 system of EEG electrode positioning. This position of the electrode ensured that the auditory cortex was stimulated (Joos et al., 2014). The reference electrode was placed on the contralateral shoulder. Each participant took part in three different sessions, carried out in 3 different days and separated by at least 24 h. Each session corresponded to one of the three conditions: “Left” (left-hemispheric stimulation), “Right” (right-hemispheric stimulation), and “Sham” (unreal stimulation: control condition). In the sham condition the electrode was placed on one of the two stimulation sites (balanced between subjects), and the current was turned off after 15 s, so that participants can feel the initial itching sensation of being stimulated, without undergoing effective modulation of cognitive functions by tDCS (Gandiga et al., 2006). The order of three sessions was counterbalanced across subjects.

**Figure 1:**
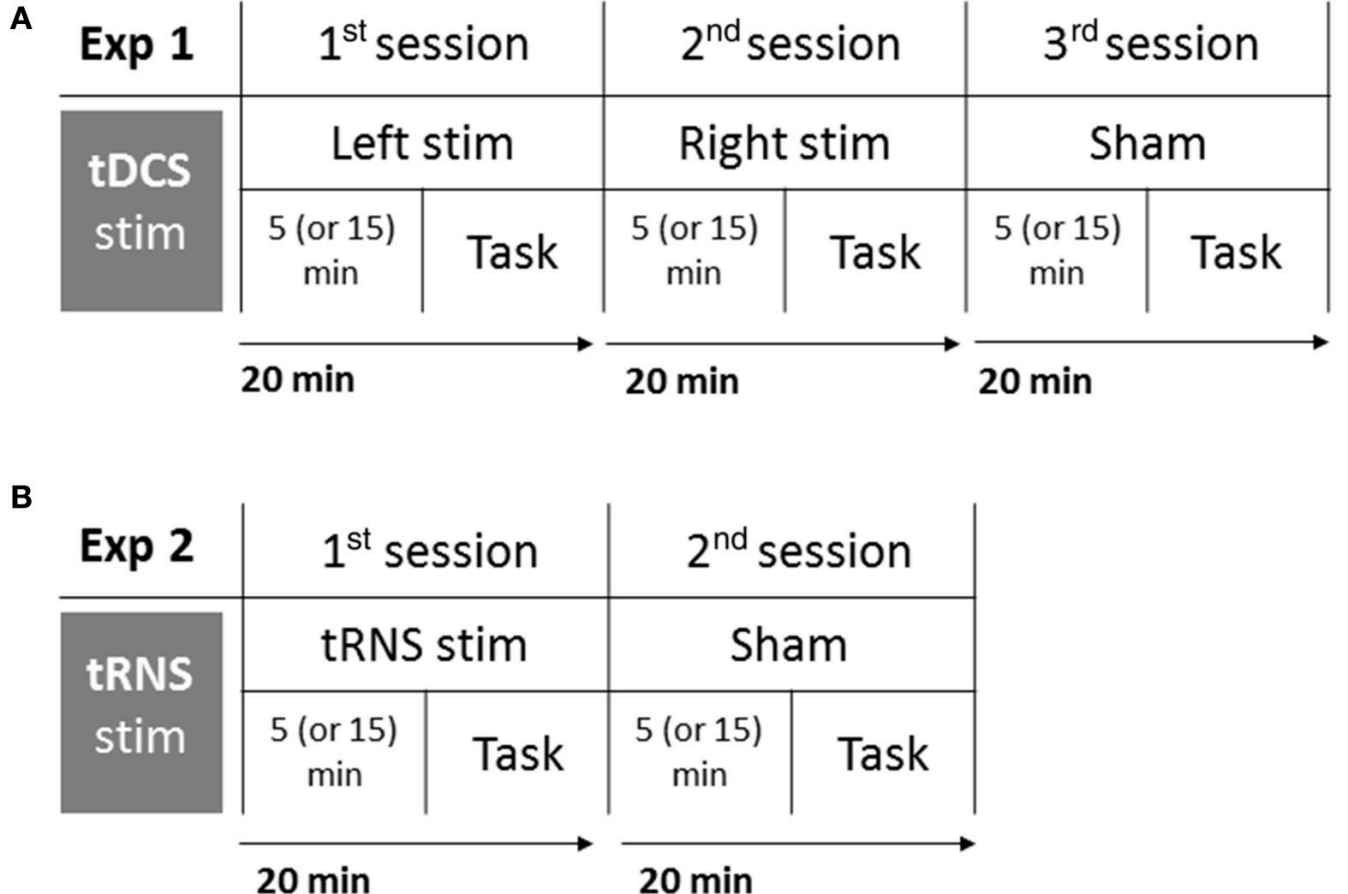
Experimental procedure of Experiment 1: tDCS **(A)**, and Experiment 2: tRNS **(B)**. Participants completed three sessions in Experiment 1 (Left stimulation, Right stimulation, Sham), and two sessions in Experiment 2 (tRNS, Sham). Each session lasted 20 min: after 5 or 15 min from the beginning of stimulation, participants performed the task. Order of sessions and interval of stimulation before the task were balanced among participants.

Participants were tested in isolation, they comfortably sat at a distance of ~80 cm from the computer screen, in a dark and silent room, and they were informed that they could stop the experiment at any time by asking the experimenter who stood behind them. The task lasted about 5 min and it was completed online (i.e., during the stimulation). In order to control for the possible effect of the stimulation duration, an half of the sample was instructed to start the task after 5 min from the beginning of the stimulation, and the other participants were required to start after 15 min. In order to keep a high attention level of participants and to make unclear which was the main task, all participants were involved in the main auditory task (Deutsch's illusion) as well as in a multimodal audiovisual filler task (not analyzed). Participants who started the auditory task after 5 min of stimulation were required to carry out the audiovisual task during the last 10 min of stimulation (after the auditory task), whereas participants who started the auditory task after 15 min of stimulation were required to carry out the audiovisual task after 5 min of stimulation (before the auditory task). The time of stimulation before the beginning of the task was randomized among participants, and—within each participant—among sessions (Left, Right, Sham). The procedure is shown in Figure 1A.

At the end of the whole procedure (after the third session), during debriefing, participants were asked whether they noticed something in particular during any session (i.e., if they believed to receive real stimulation or sham during each session), and none of the participants reported any difference between the real stimulation and the sham condition.

#### Stimuli and procedure

**Figure 2:**
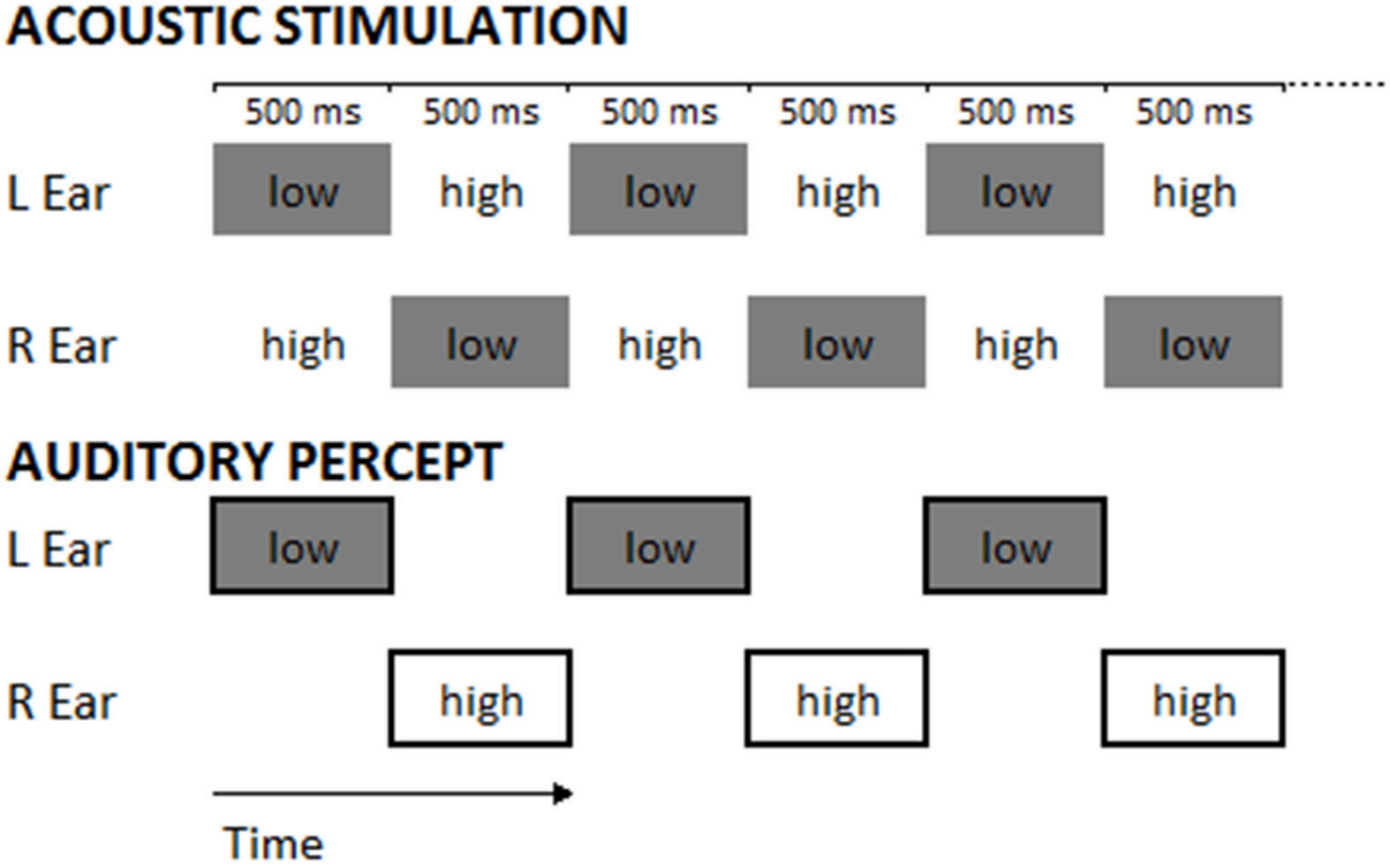
The upper panel represents the stimuli constituting the experimental trials (numbers indicate tone frequencies in Hz; in each trial 20 dichotic stimuli were presented). The lower panel represents the typical perception of a participant during the Deutsch's illusion. Low and High refer to the pitch of tones (400-Hz tone: low-pitched tone; 800-Hz tone: high-pitched tone).

The task consisted in the classical paradigm of the “Deutsch illusion” (Deutsch, 1974a,b; Deutsch, 1975, 1978): a couple of tones was presented, each in one ear (dichotic presentation), one of 400 Hz and the other of 800 Hz frequency. Each tone was a sinusoid lasting 500 ms and presenting an amplitude envelope with an attack of 10 ms and a decay of 490 ms. The two tones were alternately presented in the two ears without interstimulus interval, constituting a sequence of 20 dichotic stimuli for each trial, which lasted 10 s (see Figure 2). Half of the sequences started with the 800 Hz-tone in the left and the 400 Hz-tone in the right ear, the other half started with the 400 Hz-tone in the left and the 800 Hz tone in the right ear; this difference was balanced among participants and sessions. Sequences of sounds were delivered through headphones at an intensity of 70 dBA.

In each of the three stimulation sessions (Left, Right, Sham), eight sequences were presented (4 starting with the 400 Hz-tone and 4 starting with the 800 Hz-tone in the left ear). At the end of each sequence, participants were asked to judge if they perceived the “typical” Deutsch's illusion or otherwise. In the first case participants were asked to judge the last sound heard, choosing from a list of four possible responses (Windows Option buttons): (1) low-pitched tone in the left ear; (2) low-pitched tone in the right ear; (3) high-pitched tone in the left ear; (4) high-pitched tone in the right ear. If participants did not perceive the sequence of tones in an illusory manner they could choose from the following responses: (5) a single sound (a sound that does or does not oscillate between left and right ear); (6) more sounds (overlapping sounds, that could be perceived at the same time in both ears); (7) other (other types of percepts, not included in the previous categories). In each trial, after the presentation of the sequence of tones, the list of seven responses was presented in the center of the computer screen and participants were asked to chose which of them better defined the last sound they heard, by using the mouse. After clicking on the corresponding button, subjects pressed another button (by using the mouse) to move to the next trial. This means that before pressing the button which allows participants to shift to the next trial, they could change the response given.

The list of the responses was shown to participants before the beginning of the task and they were presented together with a high- and a low-pitched tone, in order to clarify the definition of “high-pitched tone” and “low-pitched tone” and to ensure the understanding of all of the response alternatives. Moreover, in order to become familiar with the task, before the experimental trials participants were presented with two control trials and they were asked to verbally describe their percept. Finally, they were asked to take all the time they needed in each trial to accurately select the response which better described their perception. All participants were instructed to provide the responses using the right hand.

#### Data analysis

The frequency of each response type (1–7) obtained from each participant was transformed into percentage. The mean percentage was then computed for each of the three sessions (Left, Right, Sham) and for each experimental group (anodal stimulation, cathodal stimulation), separately. Participants perceiving the illusion (low/high-pitched tone in the left/right ear) in <50% of the trials in the Sham session were excluded from further analysis (one participant in the anodal group, and three participants in the cathodal group). Mean percentages and standard errors for each category of response from the 56 remaining participants are shown in Table 1A.

**Table 1:** Mean percentages (and standard errors) for each category of responses: 1 = low-pitched tone in the left ear; 2 = low-pitched tone in the right ear; 3 = high-pitched tone in the left ear; 4 = high-pitched tone in the right ear; 5 = a single sound; 6 = more sounds; 7 = other.

		**Low/Left**	**Low/Right**	**High/Left**	**High/Right**	**Single**	**More**	**Other**	**ILL**	**vs. 57%**	**NO-ILL**	**vs. 43%**
(A) tDCS: Anodal group	Left	21.98 (4.00)	23.71 (3.64)	23.71 (4.18)	24.57 (3.85)	3.02 (2.16)	2.15 (1.40)	0.86 (0.60)	93.96 (3.92)	16.14	6.03 (1.20)	−16.14
	Right	20.26 (3.42)	25 (3.45)	22.41 (3.58)	23.71 (3.64)	4.31 (1.42)	1.72 (1.35)	2.59 (2.59)	91.38 (3.52)	10.27	8.62 (1.79)	−10.27
	Sham	21.98 (3.86)	28.45 (3.92)	23.71 (3.58)	21.12 (3.57)	2.59 (1.30)	1.72 (1.02)	0.43 (0.43)	95.26 (3.73)	24.35	4.74 (0.92)	−24.35
tDCS: Cathodal group	Left	20.37 (3.60)	28.70 (3.82)	26.85 (3.57)	19.91 (3.15)	2.31 (1.16)	1.85 (0.87)	0.00	95.83 (3.54)	23.76	4.16 (0.68)	−23.76
	Right	18.05 (2.61)	32.87 (3.21)	28.24 (3.17)	16.67 (3.34)	2.31 (1.16)	1.85 (1.85)	0.00	95.83 (3.08)	18.41	4.16 (1.00)	−18.41
	Sham	20.83 (3.40)	28.24 (3.50)	25.92 (2.82)	19.44 (3.42)	3.70 (1.61)	0.46 (0.46)	1.39 (1.02)	94.44 (3.29)	20.72	5.55 (1.03)	−20.72
(B) tRNS	tRNS	23.78 (3.32)	22.26 (3.13)	23.78 (3.14)	21.34 (2.94)	4.27 (1.42)	3.96 (1.66)	0.61 (0.61)	91.16 (3.13)	16.59	8.84 (1.23)	−16.59
	Sham	24.08 (2.81)	21.04 (3.22)	25.61 (3.32)	24.69 (3.42)	2.74 (1.11)	1.83 (0.82)	0.00	95.43 (3.19)	25.67	4.57 (0.65)	−25.67

*The upper panel represents the results collected in Experiment 1 (tDCS: Anodal/Cathodal stimulation applied over the Left auditory cortex, Right auditory cortex, and Sham condition). The lower panel represents the results collected in Experiment 2 (hf-tRNS and Sham condition). The rightmost columns represent the sum of the illusory responses (ILL: response categories from 1 to 4) and of the remaining non-illusory responses (NO-ILL: response categories from 5 to 7) in each stimulation condition, and the respective t-values when ILL was compared to the reference value of 57% and NO-ILL was compared to the reference value of 43% (Anodal tDCS: df = 28, Cathodal tDCS: df = 26, tRNS: df = 40; for all comparisons: p < 0.001)*.

The first step of the analysis was aimed at evaluating the effect of the duration of the stimulation applied before the beginning of the task on the illusory perception. Since this time changed among participants but also among the three sessions for each participant, the effect was considered in each stimulation session (Left, Right, Sham), separately. In particular, for both groups (Anodal, Cathodal), three one-way analysis of variance (ANOVAs) were carried out, using the percentage of the 4 “illusory response” categories considered together (ILL, response 1, low-pitched tone in the left ear; 2, low-pitched tone in the right ear; 3, high-pitched tone in the left ear; 4, high-pitched tone in the right ear) as the dependent variable, and the Duration of stimulation before the beginning of the task (Short: 5 min; Long: 15 min) as the between-subjects factor.

The second step of the analysis was aimed at directly evaluating the effect of the stimulation on the percentage of illusory and non-illusory responses: the percentage of responses was used as dependent variable in an ANOVA in which Stimulation (Anodal, Cathodal) was considered as between-subjects factor and Category of response (“illusory response” categories, ILL = 1, 2, 3, 4; “non-illusory response” categories, NO-ILL = 5, 6, 7) and Session (Left, Right, Sham) were considered as within-subjects factors. Furthermore, considering that there were 4 illusory response categories and 3 non-illusory response categories, the effect of Category of response was further investigated by means of exact *t*-tests: the percentage of illusory responses was compared to the probability value of illusory categories, 57% (four illusory response categories over seven possible categories), and the percentage of non-illusory responses was compared to the probability value of non-illusory categories, 43% (three non-illusory response categories over seven possible categories). Moreover, the same analysis was repeated by dividing the percentage of response categories for Stimulation (Anodal, Cathodal) and Session (Left, Right, Sham).

The last step of the analysis was aimed at assessing the possible influence of tDCS on the different illusory response categories (low/high pitch tone in the left/right ear). A further ANOVA was carried out excluding the non-illusory responses: Session (Left, Right, Sham), Perceived frequency (Low, High) and Perceived ear (Left, Right) were considered as within-subjects factors, and Stimulation (Anodal, Cathodal) was considered as between-subjects factor. The percentage of responses was the dependent variable.

### Results

The results of the first analysis were not significant, showing that the onset of stimulation before the beginning of task did not influence the perception of the illusion (see Table 2A), and thus this factor was not considered in further analyses.

**Table 2:** Results of the one-way ANOVAs in which the percentage of the illusory response categories (low/high-pitched tones in the left/right ear) was used as dependent variable, and the duration of stimulation before the beginning of the task (Short: 5 min; Long: 15 min) was used as between-subjects factor.

**(A) EXPERIMENT 1**		**5 min**	**15 min**	***F*_(1,27)_**	***P***
tDCS: Anodal group	Left	93.75 (3.97)	94.08 (2.88)	0.004	0.947
	Right	97.73 (5.31)	87.5 (4.15)	2.3	0.141
	Sham	95.00 (2.72)	95.39 (1.98)	0.003	0.955
		**5 min**	**15 min**	***F***_**(1,25)**_	***P***
tDCS: Cathodal group	Left	94.32 (2.58)	96.87 (2.14)	0.581	0.453
	Right	92.71 (3.12)	98.33 (2.79)	2	0.191
	Sham	94.32 (2.89)	94.53 (2.39)	0.003	0.955
**(B) EXPERIMENT 2**		**5 min**	**15 min**	***F***_**(1, 39)**_	***P***
tRNS	tRNS	91.87 (2.98)	90.48 (2.91)	0.113	0.739
	Sham	95.65 (2.02)	95.14 (2.29)	0.028	0.867

*The table shows the mean percentages (and standard errors) in each condition, the F-values and the p-values for Experiment 1 (tDCS: Anodal/Cathodal stimulation applied over the Left auditory cortex, Right auditory cortex, and Sham condition) in the upper panel, and for Experiment 2 (hf-tRNS and Sham condition) in the lower panel*.

The results of the second analysis revealed that only the main effect of Category of response was significant [*F*_(1, 54)_ = 1409.73, *p* < 0.001, $\eta_{p}^{2}$ = 0.96]: the percentage of illusory responses was higher than the percentage of non-illusory responses (ILL = 94.42% ± 1.54; NO-ILL = 5.58% ± 1.54). Importantly, the interaction among Category of response, Session and Stimulation was not significant [*F*_(2, 108)_ = 1.05, *p* = 0.352], showing that neither anodal nor cathodal tDCS applied on the left/right temporal cortex influenced the perception of the Deutsch's illusion.

The significant results of the *t*-tests confirmed that participants heard the illusion [ILL: *t*_(55)_ = 31.74, *p* < 0.001; NO-ILL: *t*_(55)_ = −31.74, *p* < 0.001]. This evidence was also confirmed when the percentage of response categories was considered separately for Stimulation (Anodal, Cathodal) and Session (Left, Right, Sham), both for illusory and non-illusory responses (*p* < 0.001 for all comparisons), showing that participants gave more illusory responses than expected by chance (57%) and less non-illusory responses than expected by chance (43%) in all conditions (see Table 1A).

No significant results were found in the last analysis, aimed at assessing the possible influence of tDCS on the different illusory response categories, showing that tDCS did not influence the perception of the Deutsch's illusion (see Table 1A).

## Experiment 2 (tRNS)

### Materials and methods

#### Participants

A sample of 45 healthy volunteers took part in the study (28 females, mean age: 22.67 ± 0.42). None of them took part in Experiment 1. All participants were right-handed and the mean handedness score of the sample was 64.39 (±3.23), as measured by the Edinburgh Handedness Inventory (Oldfield, 1971). Participants were enrolled if they did not show auditory impairments and no different hearing thresholds (±5 dBA) between left and right ears, as measured by an audiometric functional assessment (Brancucci et al., 2005), and if they were free from psychiatric or neurological disorders. All participants had normal or corrected-to-normal vision, and no implanted metal objects. They gave their written consent before beginning the experiment and they were informed that they could withdraw from the study at any time by asking the experimenter who was positioned behind them. The whole procedure was carried out in accordance with the principles of the Declaration of Helsinki, the protocol was approved by the Biomedical Research Ethics Committee, University of Chieti-Pescara, and participants gave written and informed consent before beginning the experiment.

#### tRNS and general procedure

Transcranial random noise stimulation (tRNS) was delivered by a battery-driven, constant current stimulator (DC-Stimulator, NeuroConn GmbH, Germany) through a pair of surface saline-soaked sponge electrodes, one measuring 5 × 9.5 cm and the other measuring 5 × 5 cm, kept firm by elastic bands. A random noise current with intensity 1.5 mA and with 0 mA offset was applied for 20 min at random frequencies ranging from 100 to 640 Hz (high frequency), according to safety guidelines (Poreisz et al., 2007), with a ramping period of 15 s both at the beginning and at the end of the stimulation.

A bilateral montage was used in order to stimulate the left and right temporal lobes, placing the electrodes between C3/T3 and C4/T4 (specifically centered on C5 and C6 sites) sites of the 10–20 EEG positioning system, thus ensuring that the auditory cortex was stimulated (Joos et al., 2014). In order to sidestep the disadvantage of having possible unwanted excitability due to cephalic montage with equally sized electrodes, and from the evidence of a better spatial resolution obtained by using electrodes with different areas due to the higher current density under the smaller electrode (Nitsche et al., 2007), the size of the electrodes was arranged in order to be asymmetrical. Thus, when the larger electrode was placed on the left hemisphere, the smaller electrode was placed on right hemisphere, and vice versa. However, since hf-tRNS with offset 0 was used, in both cases a bilateral temporal stimulation was obtained. We decided to use electrodes with different sizes as in a number of previous tRNS paradigms (e.g., Terney et al., 2008; Fertonani et al., 2011; Pirulli et al., 2013), but we did not expect to find differences between the two montages (smaller electrode over the left/right AC) because the polarity-independent stimulation used ensures a bilateral temporal stimulation.

Each participant took part in two different sessions: tRNS and Sham. In the Sham session the current was turned off after 15 s, ensuring that there was no effective current delivery after this time, and the order of sessions was counterbalanced across participants. As in Experiment 1, half of the sample was required to start the task 5 min after the beginning of the stimulation and the other half was required to start 15 min after it, in order to control for the possible effect of the prior duration of the stimulation. The stimuli and all of the other procedural details were similar to those described in Experiment 1 (see Figure 1B).

#### Data analysis

The frequency of responses was transformed into percentage for each participant. The mean percentage was then obtained for each session (tRNS and Sham). Participants who referred to perceive the illusion (low/high-pitched tone in the left/right ear) in <50% of the trials in the Sham session were excluded from further analysis (four participants). Mean percentages and standard errors for each category of responses from the remaining 41 participants are shown in Table 1B.

The same analysis as that carried out in Experiment 1 was also applied in the present Experiment. Firstly, two one-way ANOVAs were carried out in order to control for the possible effect of the duration of the stimulation applied before the beginning of the task in each stimulation session (tRNS, Sham), separately. Duration of stimulation before the beginning of the task (Short: 5 min; Long: 15 min) was used as the between-subjects factor and the percentage of the 4 “illusory response” categories considered together (ILL: low/high-pitched tones in the left/right ear) was used as the dependent variable.

In a second step of the analysis, the percentage of responses was used as dependent variable in an ANOVA in which Category of response (“illusory response” categories, ILL = 1, 2, 3, 4; “non-illusory response” categories, NO-ILL = 5, 6, 7) and Session (tRNS, Sham) were considered as within-subjects factors. Furthermore, considering that there were 4 illusory response categories and 3 non-illusory response categories, the effect of Category of response was further investigated by means of exact *t*-tests: the percentage of illusory responses was compared to the probability value of illusory categories, 57% (4 illusory response categories over 7 possible categories), and the percentage of non-illusory responses was compared to the probability value of non-illusory categories, 43% (3 non-illusory response categories over 7 possible categories). Moreover, the same analysis was repeated by dividing the percentage of response categories for Session (Stimulation, Sham).

Finally, in order to assess the possible influence of tRNS on the different illusory response categories, in the last ANOVA the percentage of responses was used as the dependent variable, and Perceived frequency (High, Low), Perceived ear (Left, Right) and Session (hf-tRNS, Sham) were considered as within-subjects factors.

### Results

The results of the one-way ANOVAs were not significant in both tRNS and Sham (see Table 2B), and thus this factor was not further considered in following analyses.

**Figure 3:**
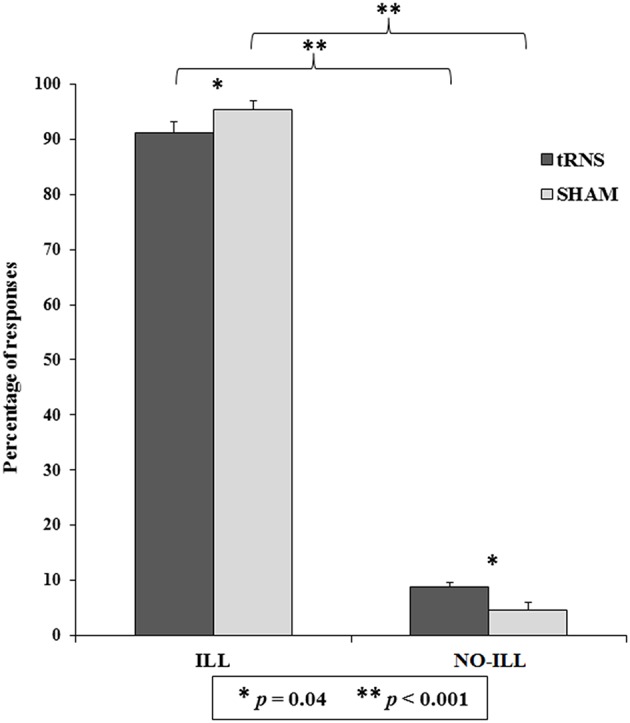
Percentage of responses indicating whether the sequence of sounds was perceived as the Deutsch's illusion (ILL) or in a non-illusory manner (NO-ILL), during the real stimulation (tRNS) and during the control condition (Sham). Asterisks show the significant comparisons and bars represent standard errors.

The second analysis revealed that the main effect of Category of response was significant [*F*_(1, 40)_ = 685.56, *p* < 0.001, $\eta_{p}^{2}$ = 0.94]: illusory responses were higher than non-illusory responses (ILL = 93.29 ± 1.29; NO-ILL = 6.71 ± 1.29). Also the interaction between Category of response and Session was significant [*F*_(1, 40)_ = 9.01, *p* = 0.005, $\eta_{p}^{2}$ = 0.18; see Table 1B], as well as all of the *post-hoc* comparisons carried out by means of Duncan test. In particular, illusory responses were higher than non-illusory responses in both tRNS and Sham (*p* < 0.001 for both comparisons), and—importantly—participants gave more illusory responses during the Sham than during tRNS (*p* = 0.040), and they also gave more non-illusory responses during tRNS than during the Sham (*p* = 0.040; see Figure 3).

The results of the *t*-tests were significant both for illusory and not-illusory responses [ILL: *t*_(40)_ = 21.95, *p* < 0.001; NO-ILL: *t*_(40)_ = −21.95, *p* < 0.001], confirming that participants heard the illusion. Similarly, the results were also significant when the percentage of response categories was considered separately for Stimulation and Sham session, both for illusory and non-illusory responses (*p* < 0.001 for all comparisons), confirming that participants gave more illusory responses than expected by chance (57%) and less non-illusory responses than expected by chance (43%) in all conditions (see Table 1B).

In the last ANOVA, only the main effect of Session was significant [*F*_(1, 40)_ = 9.01, *p* = 0.005, $\eta_{p}^{2}$ = 0.18], confirming that participants selected the illusory responses more frequently during tRNS than during sham, but no difference was found among the different illusory response categories (low/high frequency in the left/right ear).

## Discussion

The main result of the present study is that hf-tRNS applied bilaterally over the temporal cortex reduces the perception of the Deutsch's illusion. We can speculate that this evidence indirectly shows the involvement of AC in the genesis of the illusion, and that this effect can be ascribed either to the enhancement of the temporal cortex excitability or to the interference due to tRNS. The second important result of this study, however, is that this effect has been obtained only in Experiment 2, in which hf-tRNS has been used, but not in Experiment 1, in which the stimulation was delivered by means of anodal/cathodal tDCS applied over the left and right temporal cortex. A similar pattern of results has already been reported using a different auditory task, i.e., the dichotic listening paradigm. We showed that tDCS did not influence the expected right ear advantage during the dichotic presentation of consonant-vowel syllables (D'Anselmo et al., 2015), whereas bilateral hf-tRNS enhanced the expected bias compared to sham (Prete et al., under review).

The core role of the primary AC in the perception of the illusion is substantiated by neuroimaging evidence. In a magnetoencephalography study in which the neural bases of auditory consciousness were investigated using the Deutsch's illusion (Brancucci et al., 2011), it was shown that temporo-parietal areas are bilaterally involved in the conscious experience of both illusory pitch (low/high frequency) and illusory side (left/right ear) of tones. Specifically, besides the activity in frontal areas, a bilateral network including the Heschl's gyrus and the middle temporal gyrus was activated during the experience of low/high pitched tones, and a following bilateral activity in the inferior parietal lobe accompanied the perception of the side. Of note, the dimension of the electrodes we used in present study does not allow us to exclude that the stimulation also reaches the inferior parietal lobe. Similar results were observed in an fMRI study in which a variant of the Deutsch's illusion was used, made necessary because of the low temporal resolution of fMRI (Brancucci et al., 2016). By using psychophysics and EEG, Mehta et al. (2016) found that the perception of the Deutsch's illusion can be modulated by manipulating the attentional focus and that it is based upon mechanisms involving auditory stream segregation. In a following study, Mehta et al. (2017) suggested that the illusion can be due to a misattribution of time across perceptual streams, rather than a misattribution of location within a stream. Finally, the involvement of AC in the (non-illusory) perception of sequences of tones alternating in frequency (400–800 Hz) and/or side (left/right ear) has been also shown in an EEG study (Brancucci et al., 2012). All these findings suggest that AC is involved in the perception of the Deutsch's illusion, substantiating the effectiveness of the present electrical stimulation.

The present results reveal on one hand that hf-tRNS applied bilaterally over the AC decreases the classical perception of the Deutsch's illusion, and on the other hand that neither anodal nor cathodal tDCS applied unilaterally over the left and right AC influences the same task. In line with the present results Vanneste et al. (2013) found that tRNS induced stronger effects with respect to both tDCS and transcranial alternating current stimulation (tACS) on the tinnitus loudness (and related distress), when tES is applied on the AC bilaterally.

Moreover, it has to be highlighted that besides the different tES techniques we used in the two studies, also the electrode montage changed between one another: we used an extracephalic montage in the tDCS study, and a bilateral cephalic montage in the tRNS study. This difference is due to the fact that in the first study we aimed at investigating the effects of anodal/cathodal tDCS applied over the left and right AC, and thus we decided to place the reference electrode outside of the head, in order to avoid possible confounding effects of the reference electrode with an opposite current polarity. In the second study, the use of tRNS with offset 0 prevented this issue, due to the fact that this type of stimulation is not polarity-dependent. For this reason, we decided to exploit a bilateral montage with the aim to increase the AC stimulation. The bilateral montage itself can be intended as a possible reason for the stronger effects elicited by hf-tRNS on the perception of the Deutsch's illusion, not only because both left and right AC were simultaneously stimulated, but also because it has been shown that shorter inter-electrodes distance enhances the effect of tES (Moliadze et al., 2010). Moreover, it has to be noticed that in the present study only eight trials were used in each session. The low number of repetitions could be seen as a limitation of the study, but we preferred to avoid a higher number of repetitions in order to avoid participants to become anchored to one response. In fact, the fact that participants were presented more times with the same stimuli and the same responses could lead to possible response biases.

The present results, taken together with previous evidence, suggest that tRNS is efficient in affecting auditory processing, whereas tDCS seems to be less useful in this regard. Nevertheless, the methodological differences between the two studies here described prevent us to draw conclusions about a direct comparison between the two techniques, and further studies are needed to clarify the effects of specific manipulations (e.g., current intensity, electrodes position and distance, and so on). This study could be intended as a pioneering evidence of the effects of tES on a specific auditory illusion, since only a few studies until today have explored the effects of tES on deceptive perception. For instance, it has been shown that tDCS applied over the primary visual cortex, but not over the pre-motor cortex, decreases the visual illusion of motion in the “two-thirds power law” illusion (Scocchia et al., 2015). Similarly, Strüber et al. (2014) found that 40 Hz tACS applied over occipito-parietal cortex bilaterally influences the perception of bistable apparent motion stimuli. Moreover, tDCS applied over the temporal cortex decreases the audio-visual illusion known as “McGurk illusion” (whereas the illusory percept is enhanced by the stimulation applied over the multisensory parietal cortex; Marques et al., 2014), as well as it can influence also the “sound-induced flash illusion” (Bolognini et al., 2011). Despite these few investigations on the tDCS effects concerning perceptual illusions, to our knowledge no evidences are yet collected concerning illusions in the pure auditory domain. Our study not only is the first one in this direction, but also allows us an indirect comparison between anodal/cathodal tDCS and hf-tRNS. The finding of a relationship between AC stimulation and the Deutsch's illusion confirms on one hand the link between this cerebral substrate and the illusory perception, confirming previous neuroimaging evidence (Brancucci et al., 2011, 2012, 2016), and on the other hand it confirms the more general involvement of primary sensory areas in the genesis of perceptual illusions (e.g., Bolognini et al., 2011; Marques et al., 2014; Scocchia et al., 2015). Finally, although more studies are needed in this regard, the present results suggest that hf-tRNS is efficient in modulating the perception of an auditory illusion when applied over the AC, whereas both anodal and cathodal tDCS seems to be less efficient in AC modulation.

## Author contributions

AB designed the paradigm, GP and AD administered the task and wrote the draft, GP analyzed the data, AB and LT revised the manuscript.

### Conflict of interest statement

The authors declare that the research was conducted in the absence of any commercial or financial relationships that could be construed as a potential conflict of interest
